# Emetic Response to T-2 Toxin Correspond to Secretion of Glucagon-like Peptide-_17__–36_ Amide and Glucose-Dependent Insulinotropic Polypeptide

**DOI:** 10.3390/toxins14060389

**Published:** 2022-06-02

**Authors:** Jie Zhang, Tushuai Li, Qinghua Wu, Zihui Qin, Ben Wei, Ran Wu, Xinyi Guo, Huiping Xiao, Wenda Wu

**Affiliations:** 1 School of Biology and Food Engineering, Changshu Institute of Technology, Suzhou 215500, China; zhangj23@cslg.edu.cn; 2 Center for Clinical Mass Spectrometry, School of Pharmaceutical Sciences, Soochow University, Jiangsu 215123, China; 3 MOE Joint International Research Laboratory of Animal Health and Food Safety, College of Veterinary Medicine, Nanjing Agricultural University, Nanjing 210095, China; 2019107104@njau.edu.cn (Z.Q.); 2020107103@stu.njau.edu.cn (B.W.); wuranvicky@163.com (R.W.); 2021107103@stu.njau.edu.cn (X.G.); huiping0729@163.com (H.X.); 4 Wuxi Medical College, Jiangnan University, Wuxi 214122, China; tushuaili@jiangnan.edu.cn; 5 College of Life Science, Yangtze University, Jingzhou 434025, China; wqh212@hotmail.com; 6 Department of Chemistry, Faculty of Science, University of Hradec Kralove, 50003 Hradec Kralove, Czech Republic

**Keywords:** T-2 toxin, emesis, brain–gut peptide, calcium-sensing receptor, transient receptor potential channel

## Abstract

The T-2 toxin, a major secondary metabolite of *Fusarium Gramineae*, is considered a great risk to humans and animals due to its toxicity, such as inducing emesis. The mechanism of emesis is a complex signal involving an imbalance of hormones and neurotransmitters, as well as activity of visceral afferent neurons. The T-2 toxin has been proven to induce emesis and possess the capacity to elevate expressions of intestinal hormones glucagon-like peptide-_1__7__–36_ (GLP-1) and glucose-dependent insulinotropic polypeptide (GIP), both of which are important emetic factors. In addition, the activation of calcium-sensitive receptor (CaSR) and transient receptor potential (TRP) channels are engaged in intestinal hormone release. However, it is unknown whether hormones GLP-1 and GIP mediate T-2 toxin-induced emetic response through activating CaSR and TRP channels. To further assess the mechanism of T-2 toxin-induced emesis, we studied the hypothesis that T-2 toxin-caused emetic response and intestinal hormones GLP-1 and GIP released in mink are associated with activating calcium transduction. Following oral gavage and intraperitoneal injection T-2 toxin, emetic responses were observed in a dose-dependent manner, which notably corresponded to the secretion of GLP-1 and GIP, and were suppressed by pretreatment with respective antagonist Exending_9–39_ and Pro3GIP. Additional research found that NPS-2143 (NPS) and ruthenium red (RR), respective antagonists of CaSR and TRP channels, dramatically inhibited both T-2 toxin-induced emesis response and the expression of plasma GLP-1 and GIP. According to these data, we observed that T-2 toxin-induced emetic response corresponds to secretion of GLP-1 and GIP via calcium transduction.

## 1. Introduction

Trichothecene mycotoxins are secondary metabolites of various *Fusarium*, which frequently germinate in warm and humid weather and generally contaminate a variety of crops such as soybean, corn, and wheat, posing immense risk to humans and animals [1,2,3]. Trichothecene mycotoxins have been observed to induce a battery of acute and chronic adverse effects, including anorexia, emesis, gut intestinal injury, neuroendocrine dysfunction, diarrhea, and growth retardation in mycotoxin-exposed animals [4,5,6,7,8]. Among the trichothecene mycotoxins, T-2 toxin is considered the most poisonous, though the distribution of T-2 is not as widespread as that of deoxynivalenol (DON), another notable trichothecene mycotoxin [9,10]. Due to the powerful toxicity, the European Food Safety Authority restricts the sum of T-2 and HT-2 toxin to 100 ng/kg body weight (BW) as a maximum tolerable daily intake [11].

Emesis is ordinarily deemed to be a protective reflex against food poisoning; however, severe vomiting reactions may disrupt normal nutrient, water, and electrolyte balances, resulting in adverse health effects [12,13]. Emesis is regulated by the central pattern generator (CPG), which is considered a “vomiting center,” and the CPG concertedly balances expressions of hormones and neurotransmitters as well as the activity of visceral afferent neurons to mediate emesis [14,15]. Hormones and neurotransmitters are two critical factor groups involved in regulating feeding behavior and have been observed participating in trichothecene mycotoxin-induced anorexia. For example, DON and T-2 toxin upregulate the release of plasma hormone peptide YY_3–36_ (PYY_3–36_), glucagon-like peptide-1_7–36_ (GLP-1), glucose-dependent insulinotropic polypeptide (GIP) and cholecystokinin (CCK), as well as neurotransmitter 5-hydroxytryptamine (5-HT) and substance P in experimental animals, leading to anorexic responses [6,16,17,18]. In addition, trichothecene mycotoxins, especially DON, possess the capacity to elicit emetic response, which corresponded to elevated plasma hormone PYY_3–36_ and neurotransmitter 5-HT in experimental animals [19]. Analogous results were observed in T-2 toxin treated minks following both intraperitoneal (IP) administration and oral gavage as well [5]. The data demonstrate that T-2 toxin have the capacity to induce emesis in treated animals; however, the mechanism is still unclear.

GLP-1 and GIP are two critical hormones in regulating feeding behavior. Endogenous GLP-1 is generated by intestinal endocrine L cells in the gut and plays a critical role in the inhibition of food consumption and the delay in gastric emptying [20]. GLP-1 specifically binds G-protein-coupled GLP-1 receptors (GLP-1R), which are located in the hypothalamus, which is involved in the regulation of feeding behavior and glucose homoeostasis, and in the brainstem, which is involved in the mechanisms of emesis, playing biological roles [21,22,23,24]. GIP is synthesized and released by K cells in the intestinal epithelium, responding to nutrient ingestion and active anorexigenic signals to restrict appetite and food ingestion [25]. GIP exerts its effect by specifically activating the GIP receptor (GIPR), and the antagonist Pro3GIP markedly elevated blood glucose levels 0.5 and 1 h post feeding in 18 h fasted mice, which indicates that GIP plays a role in anorexigenic signal regulation [26,27]. These two hormones are proven to mediate anorexic inductions triggered by trichothecene mycotoxin and can be antagonized by respective receptor inhibitors [6,16,17,28]. Nevertheless, the data about GLP-1 and GIP involved in T-2 toxin-caused emesis have rarely been investigated.

Calcium-sensitive receptor (CaSR), a G-protein coupled receptor, and transient receptor potential (TRP) channels are the main Ca^2+^ sensor and are expressed in a variety of tissues, including the gastrointestinal tract. The expression of CaSR and TRP channels on intestinal endocrine cells suggests that CaSR and TRP ankyrin-1 (TRPA1) can act as chemosensors of intestinal hormone release [29,30]. CaSR is observed to play a vital role in L-tryptophan-mediated synthesis and release of GLP-1 and GIP, which can be repressed by CaSR antagonist NPS-2143 (NPS), leading to downregulation of downstream signal molecules protein kinase C and inositol trisphosphate receptor [29,31]. In addition, specific co-localization on GLP-1 and TRP channels indicate that the latter are involved in release of the former [30]. Our previous studies have demonstrated that the activations of CaSR and TRP channels are involved in DON-induced anorexia and emesis induction [19,32]. However, whether T-2 toxin-induced emetic response is correlated to the activation of CaSR and TRP channels, leading to GLP-1 and GIP release in the gastrointestinal tract, remains unclear.

The objective of the present study was to validate the hypothesis that T-2 toxin-induced emetic response and gastrointestinal hormones GIP and GLP-1 release are correlated to the activation of calcium transduction.

## 2. Results

### 2.1. Emetic Potencies of T-2 Toxin Following Oral and IP Dosing

The emetic potencies of oral and IP dosing with T-2 toxin were exhibited (Table 1). After both oral and IP treatment, 0.002 and 0.01 mg/kg bw T-2 toxin displayed no effect. Following oral exposure, 0.05 mg/kg bw was the lowest emetic dose of T-2 toxin, to which 80% of the minks responded. When the minks were orally treated with 0.25 mg/kg bw T-2 toxin, all of them vomited. The vomiting occurred within 30 min and lasted for more than 2 h. Upon IP injection, 0.05 mg/kg bw was also the lowest emetic dose of T-2 toxin, to which 40% of the minks responded. When the dose of T-2 toxin was increased to 0.25 mg/kg bw, all the minks vomited. The emetic episodes happened at about 30 min and lasted for more than 3 h.

### 2.2. T-2 Toxin-Induced Emetic Effect Corresponds to Elevation of GLP-1 and GIP

**Figure 1:**
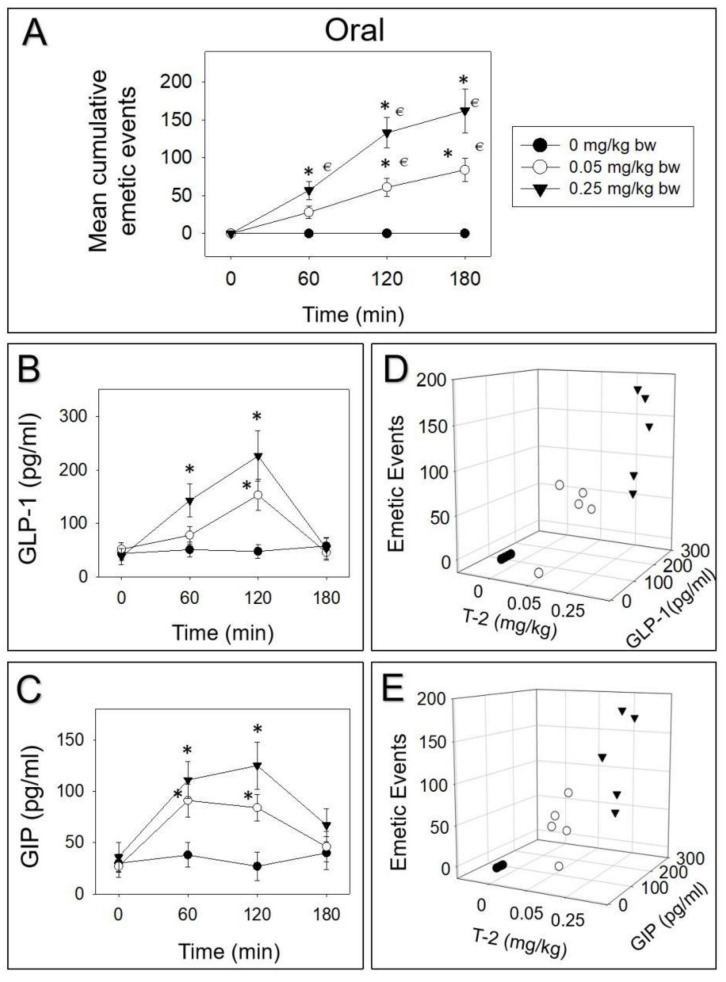
Emetic response corresponds to elevation of GLP-1 and GIP by oral exposure to T-2 toxin. (**A**) Mean cumulative emetic events in minks following oral exposure to T-2 toxin. Kinetics of T-2 toxin-induced (**B**) GLP-1 and (**C**) GIP elevation in plasma. Relationship between emetic events and (**D**) GLP-1 and (**E**) GIP levels at 120 min. Data are mean ± SEM (*n* = 5/group). Two-way ANOVA using the Holm–Sidak test was used to analyze significant differences in mean cumulative emetic events and kinetics of GLP-1 and GIP in minks. Symbols: * indicates a statistically significant difference in mean cumulative emetic events and GLP-1 or GIP concentration relative to the control at a specific time point (*p* < 0.05). € indicates a statistically significant difference in mean cumulative emetic events relative to the 0 min time point (*p* < 0.05). The Spearman rank–order correlation coefficient was used for the correlation between emetic events and hormone levels (*p* < 0.05).

Minks were orally gavaged and IP injected with T-2 toxin, and then GLP-1 and GIP were tested over 180 min. Upon oral treatment of 0.05 or 0.25 mg/kg bw T-2 toxin, 33%, 39%, and 27% or 35%, 82%, and 18% vomiting occurred during 0–60, 60–120, and 120–180 min periods, respectively (Figure 1A). Following oral treatment of 0.25 mg/kg bw T-2 toxin, plasma GLP-1 was elevated at 60 and 120 min (Figure 1B). After 180 min, GLP-1 returned to basal level. On the other hand, 0.05 mg/kg bw T-2 toxin displayed a significant effect on GLP-1 only at 120 min. Plasma GIP increased at both 60 and 120 min by orally dosing with 0.05 or 0.25 mg/kg bw T-2 toxin (Figure 1C). Dose-dependent elevation of GIP and SP corresponded to emetic episodes triggered by oral exposure to D3G at 120 min (Figure 1D,E).

**Figure 2:**
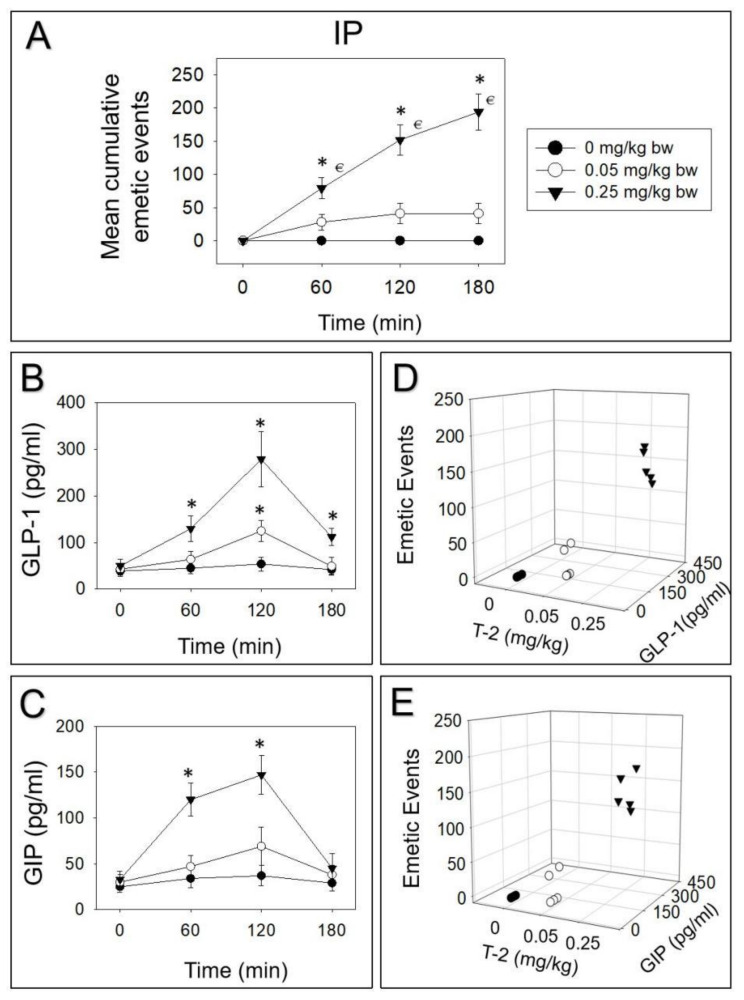
T-2 toxin-induced emetic response (**A**) corresponds to elevation of GLP-1 (**B**,**D**) and GIP (**C**,**E**) following IP exposure. Data are mean ± SEM (*n* = 5/group). Two-way ANOVA using the Holm–Sidak test was used to analyze significant differences in mean cumulative emetic events and kinetics of GLP-1 and GIP in minks. Symbols: * indicates a statistically significant difference in mean cumulative emetic events and GLP-1 or GIP concentration relative to the control at a specific time point (*p* < 0.05). € indicates a statistically significant difference in mean cumulative emetic events relative to the 0 min time point (*p* < 0.05). The Spearman rank–order correlation coefficient was used for the correlation between emetic events and hormone levels (*p* < 0.05).

IP dosing with 0.05 or 0.25 mg/kg bw T-2 toxin evoked 68%, 32%, and 0% or 40%, 38%, and 22% vomiting during 0–60, 60–120, and 120–180 min periods, respectively (Figure 2A). Plasma GLP-1 was raised at 60, 120, and 180 min by IP dosing with 0.25 mg/kg bw T-2 toxin, whereas 0.05 mg/kg bw T-2 toxin only displayed a significant effect on GLP-1 at 120 min (Figure 2B). Upon IP exposure to 0.25 mg/kg bw T-2 toxin, plasma GIP was increased at 60 and 120 min (Figure 2C). On the other hand, 0.05 mg/kg bw T-2 toxin displayed no effect on GIP. Dose-dependent elevation of GLP-1 and GIP in plasma corresponded to emetic episodes stimulated by IP exposure to T-2 toxin at 120 min (Figure 2D,E).

### 2.3. Effects of Brain–Gut Peptide Receptor Inhibitor on T-2 Toxin-Induced Emesis

**Figure 3:**
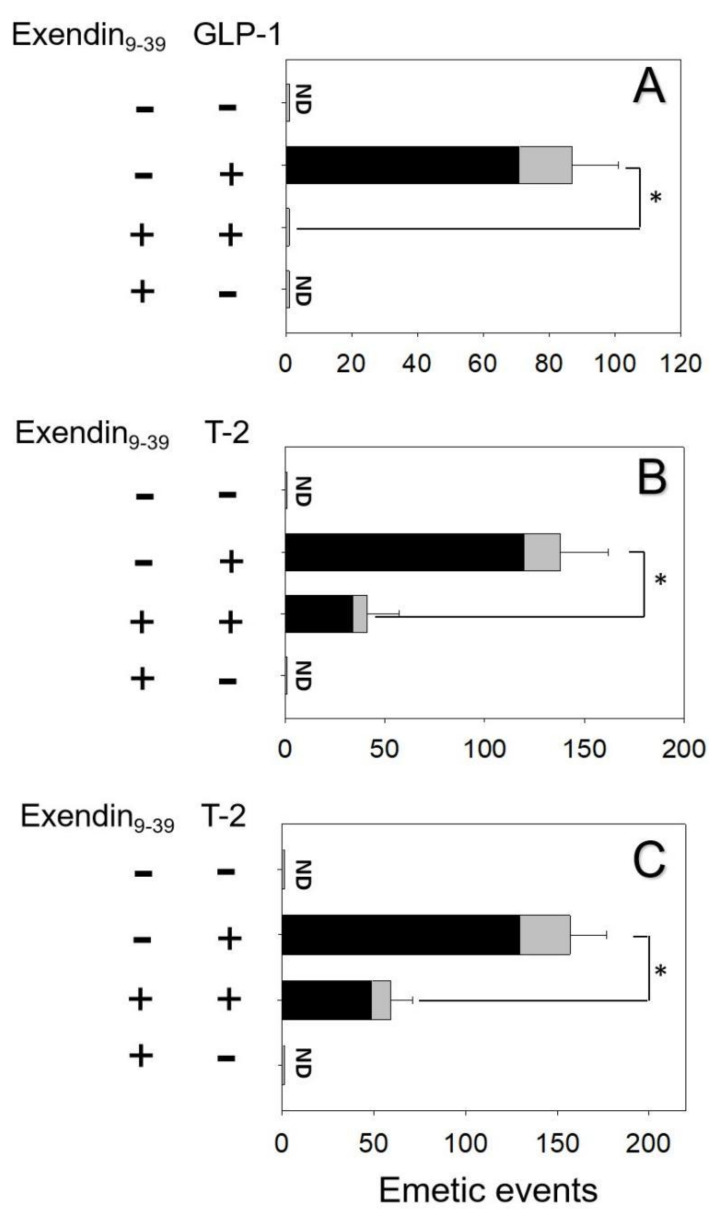
GLP−1R inhibitor Exendin_9–39_ diminished emetic episodes induced by (**A**) GLP−1 and (**B**) T−2 toxin after oral treatment and (**C**) T−2 toxin after IP treatment. Emetic episodes contain retching (black) and vomiting (gray) episodes. ND = not detected. Data represent mean ± SEM (*n* = 5/group). A one−way ANOVA using Holm–Sidak was used to assess significant differences between treatments and the respective controls. Symbols: * indicates statistically significant differences in emetic episodes (*p* < 0.05).

**Figure 4:**
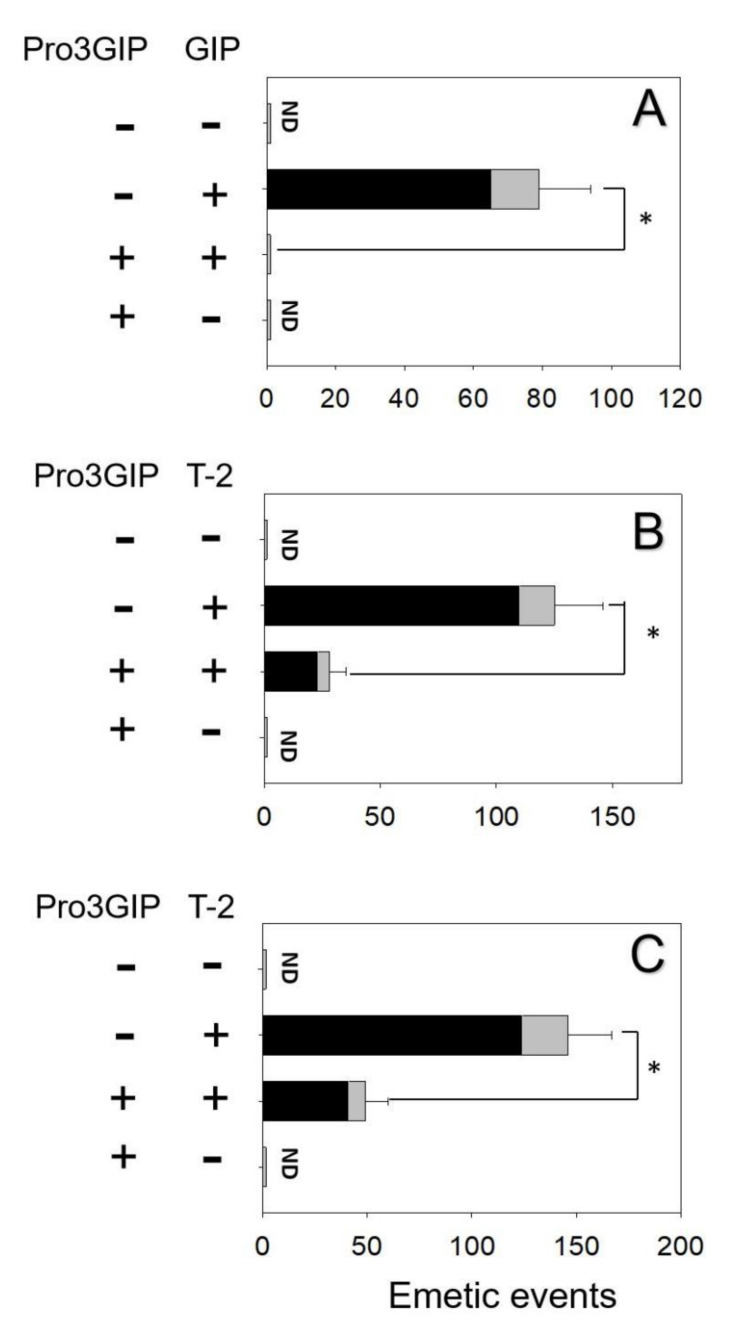
GIPR inhibitor Pro3GIP diminished emetic episodes induced by (**A**) GIP and (**B**) T−2 toxin after oral treatment and (**C**) T−2 toxin after IP treatment. Emetic episodes contain retching (black) and vomiting (gray) episodes. ND = not detected. Data represent mean ± SEM (*n* = 5/group). A one−way ANOVA using Holm–Sidak was used to assess significant differences between treatments and the respective controls. Symbols: * indicates statistically significant differences in emetic episodes (*p* < 0.05).

The effects of blocking GLP-1R in GLP-1- and T-2 toxin-induced vomiting were evaluated. Mink dosing with GLP-1 elicited 87 ± 14 emetic episodes (Figure 3A). Pretreatment of GLP-1R antagonist Exendin_9–39_ completely blocked emetic episodes induced by GLP-1. Giving Exendin9–39 alone did not lead to vomit, suggesting no functional antagonism. Animals orally and IP treated with T-2 toxin had 138 ± 24 and 157 ± 20 emetic episodes, respectively (Figure 4B,C). Receiving Exendin9–39 prior to oral and IP exposure to T-2 toxin in minks diminished % emetic episodes by 70% and 62%, respectively.

The effects of blocking the GIPR in GIP- and T-2 toxin-induced emesis were evaluated. Minks dosed with GIP displayed 79 ± 15 emetic episodes (Figure 4A). Pretreatment of GIPR inhibitor Pro3GIP completely blocked GIP-induced emetic episodes. Giving Pro3GIP alone did not lead to vomiting, suggesting no functional antagonism. Minks orally and IP dosed with T-2 toxin had 125 ± 21 and 146 ± 19 emetic episodes, respectively (Figure 4B,C). Receiving Pro3GIP prior to oral and IP exposure to T-2 toxin in mink diminished emetic episodes by 75% and 66%, respectively.

### 2.4. Roles of CaSR and TRP Channel on T-2 Toxin-Induced Emesis and Brain–Gut Peptides

**Figure 5:**
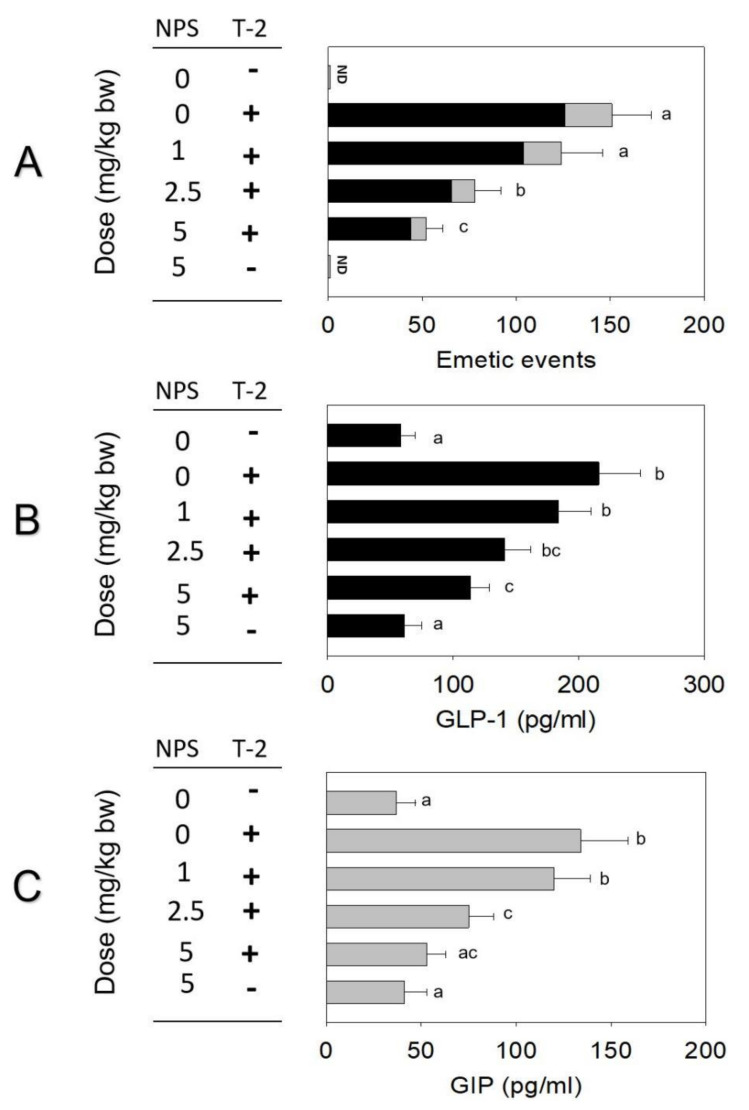
(**A**) T-2 toxin−induced emetic episodes. (**B**) GLP-1 and (**C**) GIP were dose−dependently attenuated by CaSR antagonist NPS. Emetic episodes contain retching (black) and vomiting (gray) episodes. ND = not detected. Data represent mean ± SEM (*n* = 5/group). A one-way ANOVA using Holm–Sidak was used to analyze significant differences between multiple groups. Bars without the same letter are significantly different (*p* < 0.05).

The impact of CaSR antagonist NPS on T-2 toxin-induced emesis was assessed in the minks. Oral exposure to T-2 toxin at 0.25 mg/kg bw caused 151 ± 22 emetic episodes (Figure 5A). Pre-treatment with NPS reduced emetic episodes by 18%, 48%, and 65% at 1, 2.5, and 5 mg/kg bw, respectively. Plasma GLP-1 and GIP concentrations increased significantly 2 h after oral exposure to T-2 toxin. Pre-dosing with 1, 2.5, and 5 mg/kg bw NPS reduced GLP-1 by 14%, 35%, and 47% or GIP by 10%, 44%, and 59%, respectively (Figure 5B,C).

**Figure 6:**
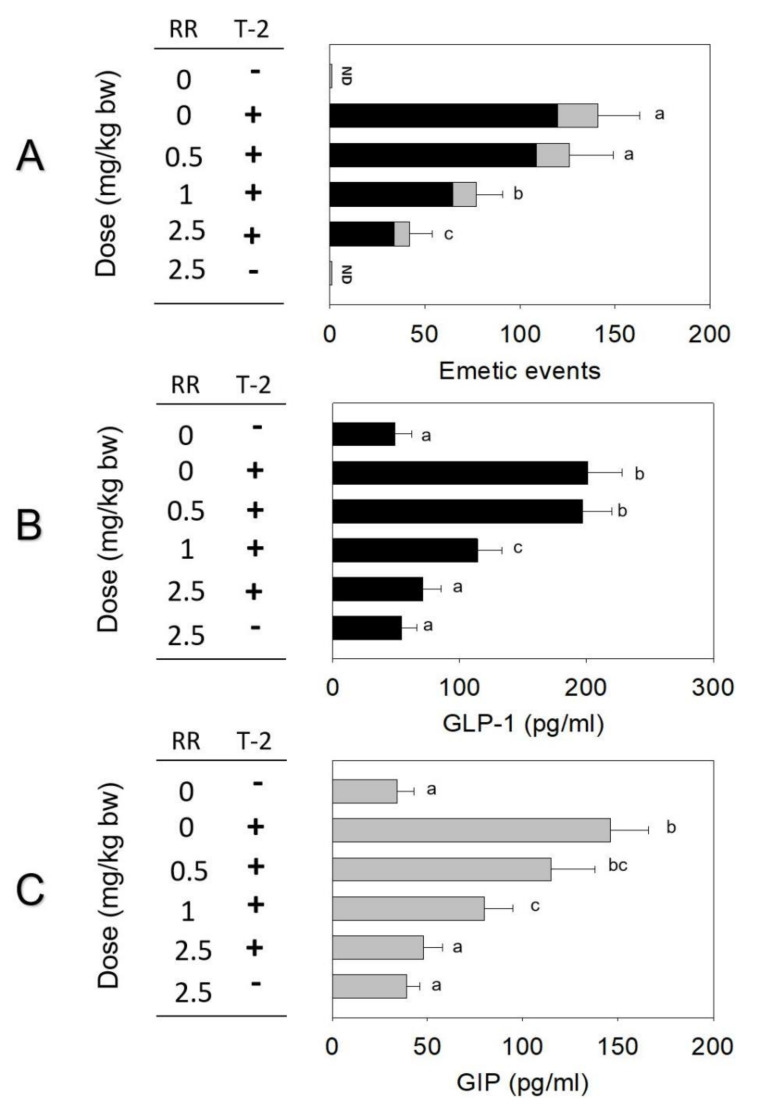
(**A**) T-2 toxin−induced emetic episodes. (**B**) GLP-1 and (**C**) GIP were dose-dependently attenuated by TRP channel antagonist RR.Emetic episodes contain retching (black) and vomiting (gray) episodes. ND = not detected. Data represent mean ± SEM (*n* = 5/group). A one-way ANOVA using Holm–Sidak was used to analyze significant differences between multiple groups. Bars without the same letter are significantly different (*p* < 0.05).

When minks were pre-dosed with TRP channel antagonist RR, emetic episodes were decreased by 11%, 45%, and 70% at 0.5, 1, and 2.5 mg/kg bw, respectively (Figure 6A). When pretreated with 0.5, 1, and 2.5 mg/kg bw RR, plasma GLP-1 or GIP declined by 2%, 42%, and 64% or 21%, 45%, and 67%, respectively (Figure 6B,C).

**Figure 7:**
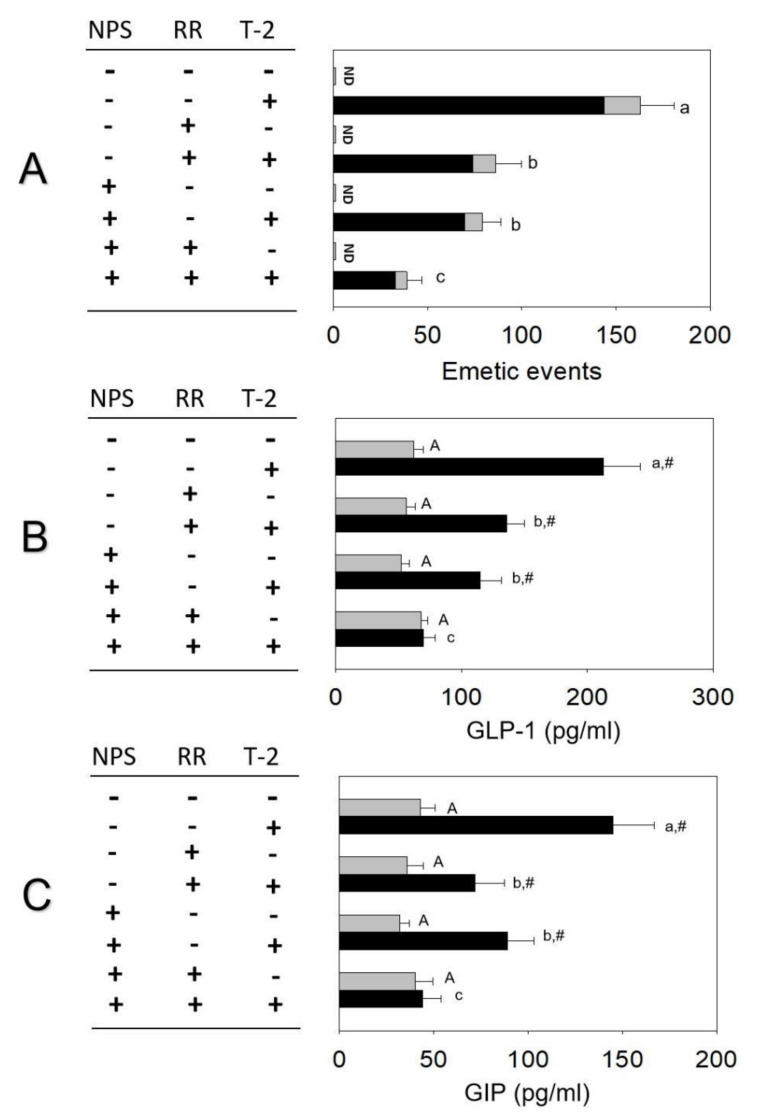
(**A**) CaSR antagonist NPS and TRP channel antagonist RR additively inhibit T-2 toxin−induced emetic episodes. (**B**) GLP-1 and (**C**) GIP in minks. Emetic episodes contain retching (black) and vomiting (gray) episodes. ND = not detected. Data represent mean ± SEM (*n* = 5/group). A one-way ANOVA using Holm–Sidak was used to analyze significant differences between multiple groups. Bars without the same letter are significantly different (*p* < 0.05). Statistical comparisons between two groups were analyzed using Student’s *t* test. The # sign indicates significant difference between VEH and the corresponding T-2 toxin−treated group (*p* < 0.05).

The combined effects of NPS and RR on T-2 toxin−induced emetic response and brain–gut peptide release are shown in Figure 7. Upon pre-dosing with 2.5 mg/kg bw NPS or 1 mg/kg bw RR, T-2 toxin−induced emetic episodes were attenuated by 52% and 47%, respectively (Figure 7A). When minks were treated with both antagonists, emetic episodes were attenuated by 73%. Plasma GLP-1 and GIP concentrations increased significantly 2 h after oral exposure to T-2 toxin. When pretreated with 2.5 mg/kg bw NPS or 1 mg/kg bw RR, T-2 toxin−induced GLP-1 was attenuated by 46% and 36%, respectively (Figure 7B). When minks were treated with both antagonists, plasma GLP-1 was attenuated by 69%. When pretreated with 2.5 mg/kg bw NPS alone, T-2 toxin−induced GIP was attenuated by 39% (Figure 7C). Minks treated with 1 mg/kg bw RR alone had a significant decrease in plasma GIP (50%). A significant decrease in plasma GIP (71%) was shown when minks were treated with both antagonists.

## 3. Discussion

The capacity of the T-2 toxin to activate the vomitory signal, a protective response to food poisoning, is a concern in relation to public health [5,33]. Former studies have demonstrated that T-2 toxin-induced emetic induction was related to PYY_3–36_ and 5-HT elevation [5]; however, two significant hormones—GLP-1 and GIP, both of which are considered vomitory factors involved in T-2 toxin-caused emesis—are indistinguishable, though they have been proven to be elevated in T-2 toxin-induced anorexia [16,24,28]. In addition, the release of GLP-1 and GIP is related to an increase in calcium transduction by activating the reactivities of CaSR and TRP. The activation of CaSR and TRP facilitates the penetrability of Ca^2+^ channel-triggered hormones release in enteroendocrine cells of the gastrointestinal tract [30,34]. Our previous study demonstrated that trichothecene mycotoxin DON-activated CaSR and TRP lead to hormone release [32]. Therefore, we hypothesized that T-2 toxin-induced emetic induction correlates to gastrointestinal hormones GIP and GLP-1 release, which are mediated by the activation of calcium transduction. In the current study, we established T-2 toxin-induced emesis in minks in a dose-dependent manner following IP administration and oral gavage methods; emesis caused by T-2 toxin was related to GIP and GLP-1 hormone elevation, and selective antagonisms of these two hormones inhibited the mycotoxin-induced emetic response. Selective antagonism of CaSR and TRP alleviated T-2 toxin-induced emesis in a dose-dependent manner, and the devitalization of CaSR and TRP impaired T-2 toxin-induced elevation of GIP and GLP-1.

The incidence, latency, duration, and frequency of emesis, the critical endpoints of vomiting efficacy, caused by T-2 toxin in minks are compatible with former research results following both IP administration and oral gavage methods [5,35]. IP administration and oral gavage of T-2 toxin at 0.25 mg/kg bw induced vomiting within 25 min and the duration was 161 and 124 min, respectively. It seems that IP-administered T-2 toxin may cause a longer duration of vomiting than the oral gavage method. The T-2 reached its maximum plasma level approximately 30 min after exposure and evoked great emesis [36]. The prolonged duration of emetic response may be attributed to T-2 toxin’s inherent ability to sustain a reaction at high toxicity or low concentrations, though the plasma half-life of T-2 is brief [37,38].

The emetic center located in the brainstem is contained within the nucleus of the solitary tract (NTS), the dorsal motor nucleus of the vagus, and the area postrema (AP) [39,40]. The AP/NTS is the chief target of vagal afferent projection from the gut, and moreover, the upregulated activity of the former neuron accompanies emesis [39,41,42]. Nevertheless, emesis regulation is a complicated system involving gut hormones, neurotransmitters, and visceral afferent neurons [14,15]. Of particular note, T-2 toxin-induced vomiting corresponds to prominent plasma levels of GLP-1 and GIP, hormones known to induce emetic action [22,43]. The possible reason for rapid onset of vomiting induced by T-2 toxin is that fenestrated capillaries permit the AP/NTS to be touched simply by circulating T-2 toxin [41,44]. Additionally, during emesis induced by 0.25 mg/kg bw T-2 toxin, the expressions of GLP-1 and GIP were upregulated and peaked at 120 min. To further assess whether GLP-1 and GIP are involved in T-2 toxin-induced emesis, we applied Exendin9–39 and Pro3GIP, which are the specific receptor antagonists of GLP-1 and GIP, respectively, and observed that T-2 toxin-induced emetic events were markedly decreased. GLP-1, which is synthesized by enteroendocrine L cells of the intestine, is released into the interstitial space and then diffuses to act on GLP-1R located on the vagal nerve endings embedded in the gut mucosa or somewhere else near the rest of the circulation, releasing the co-transmitters in the brain stem or acting on the single region AP, where mostly c-Fos-positive cells express GLP-1R to regulate feeding behavior [45,46,47,48,49,50]. Over the past two decades, GLP-1 and its analogues have been widely studied as treatments for type-2 diabetes and obesity; however, the most common side effects, including nausea and vomiting, slow down the progress of research [51,52,53]. Amazing data demonstrate that up to 6.5 million patients (50% of type-2 diabetes in Americans treated with GLP-1-based drugs) may experience nausea and emesis [51]. Coincidentally, GIPR is expressed in the AP and is involved in emesis regulation [54,55,56]. Interestingly, the latest study demonstrated that GLP-1R agonist GLP-140 administration-induced kaolin consumption, which was used to assess vomiting in laboratory rodents, could be attenuated by an infusion of GIP-532, a short-acting GIPR agonist, into the fourth ventricle (only targeting hindbrain GIPR-expressing cells) [24]. There seems to be certain correlation between GLP-1R and GIPR [24,57]. GIPR antagonist Pro3GIP in T-2 toxin-induced emesis may be mediated through insulin-dependent mechanisms [58]. Therefore, the data presented here indicate that gastrointestinal hormones GLP-1 and GIP play critical roles in T-2 toxin-induced emetic effects. Considering that endogenous GLP-1 and GIP are rapidly degraded by dipeptidyl peptidase 4 [59,60,61], intestinal incretin is unlikely to reach receptors in the brain, indicating that the mechanisms of endogenous GLP-1 and GIP in regulating emesis remain undefined.

The present data displayed here indicate that CaSR plays a critical role in T-2 toxin-induced GLP-1 and GIP release, both of which contribute to mycotoxin’s emetic effects. CaSR is expressed in various organs, including the gastrointestinal tract, to regulate energy balance [62]. The expression of CaSR on intestinal endocrine cells suggests that CaSR may impersonate a sensor of intestinal hormone release [63]. γ-[Glu](n = 1,2)-Phe/-Met/-Val upregulated the activation of CaSR and engendered Ca^2+^ mobilization, leading to GLP-1 release from intestinal neuroendocrine tumor STC-1 cells [64]. After the acute intraduodenal administration of L-tryptophan, GLP-1 level in plasma was remarkably elevated and could be attenuated by CaSR antagonist NPS [31]. Zhao et al. observed that GIP secretion was induced by L-tryptophan in swine duodenum involved in CaSR activation [29]. Wang et al. found that intraduodenal infusion of soybean protein hydrolysate restrained temporary feed consumption in pigs and advanced GIP release, which is related to the activation of duodenum CaSR [65]. T-2 toxin seems to play an analogous role to these L-amino acids to activate CaSR and induce Ca^2+^ mobilization, leading to GLP-1 release from the intestinal neuroendocrine. This is in accordance with our previous research that DON selectively activated CaSR and elevated hormones CCK and PYY_3–36_ to mediate anorexia [32]. However, NPS did not antagonize CaSR-mediated GLP-1 and GIP elevation in an intraduodenal infusion in pigs [65]. The difference of stimulant, dosage, and experimental animal species may account for the disagreement.

TRP is another critical factor to mediate Ca^2+^ transduction. The distribution of TRP channels is found in chemosensory cells throughout the digestive, respiratory, and olfactory systems, and TRP channels have well-established roles in the regulation of gastrointestinal motility and absorption [66]. Microarray analysis was applied to analyze the expression of mRNA-encoding TRP channels in murine K- and L-cell populations and in STC-1 cell lines. TRPA1, TRP channel 1 (TRPC1), TRPC3, TRPC4, TRPC5, and TRP melastatin-subfamily member 7 were found to have high expression in enteroendocrine cell populations [67]. The TRPA1 agonist activated GLP-1 synthesis in primary murine intestinal cultures and could be abolished by pharmacological TRPA1 inhibition [67]. Very low TRP vanilloid 1 (TRPV1) mRNA levels in all the tested cells were observed by Emery; however, Wang et al. observed TRPV1 immunohistochemically in STC-1 cells [68]. Elevation of GLP-1 and insulin was observed following capsaicin treatment by gastric gavage in wild-type mice but not in TRPV1 knockout mice, and similar results were observed in capsaicin-treated STC-1 cells [68]. Therefore, which TRP subtype it is that affects the Ca^2+^ transduction to mediate GLP-1 release needs further research. It is worth noting that nearly no data demonstrate the connection between GIP release and TRP channel activation. In the present study, we first illustrated that activated TRP channels mediate GIP synthesis and release while the specific antagonist RR attenuates GIP release. In our previous studies, we found that TRPA1 involved in DON-induced hormones such as GLP-1 release from the STC-1 enteroendocrine cell model [19,32,69]. T-2 toxin-triggered GLP-1 and GIP release has been observed in T-2 toxin-induced anorexia as well. These results demonstrate that TRP channels are involved in trichothecene mycotoxin-induced hormone release. The combined utilization of antagonists NPS and RR present synergistic effects, and the data further illustrate the hypothesis that T-2 toxin regulates calcium transduction to lead to GLP-1 and GIP hormone release.

## 4. Conclusions

To summarize, the data presented herein show that T-2 toxin induces emetic response as well as plasma GLP-1 and GIP elevation by activating CaSR and TRP channel, leading to continuous calcium transduction for a period of time. Former studies indicate that T-2 toxin has the capacity to elicit intestinal hormone synthesis and release, leading to emesis and anorexia, which are pose great risk to public health [5,6,16]. CaSR and TRP channels have been observed to function as critical factors in mycotoxin-induced abnormal feeding behavior [32], and subsequent investigations should concentrate on how CaSR and TRP channels are involved in mediating trichothecene-induced hormone release from intestinal enteroendocrine cells. Further understanding the mechanism of T-2 toxin-induced emesis can potentially lead to better prevention and treatment strategies in animals and humans [70].

## 5. Materials and Methods

### 5.1. Animal and Reagent

Minks (1–2 years old, female) were obtained from Far East Breeding Co., Ltd. (China, Shandong, Weifang) and housed in individual cages with a 12 h light/dark cycle, 20–24 °C, and 30–70% humidity. Guidelines for animal study were established by the Institutional Animal Care and Use Committee at Nanjing Agricultural University (Certification No: SYXK (Su) 2011-0036). T-2 toxin was purified from wheat as previously described [6] and the purity (>96%) was measured by HPLC-tandem mass spectrometry (HPLC-MS/MS). GLP-1, GIP, and receptor inhibitors Exendin_9–39_ and Pro3GIP were obtained and prepared according to prior studies [71]. NPS-2143 (NPS) and RR were purchased and prepared according to previous studies [19,32].

### 5.2. Experimental Design

Study 1: Emetic potencies of T-2 toxin following oral and IP dosing

Minks (*n* = 5/group) first fasted 24 h, and then were allowed to eat feed for 30 min before the experiment. Then, the minks was orally gavaged or IP injected with 0, 0.002, 0.01, 0.05, and 0.25 mg/kg bw T-2 toxin using a sterile stainless-steel gavage tube (16-G, 5-cm) or using a sterile needle (20-G, 2.54-cm), respectively. After that, emesis in the minks was monitored over the subsequent 6 h. Each individual retch or vomit was counted as described previously [5].

Study 2: Effect of T-2 toxin on brain–gut peptides

After fasting and re-feeding as previously mentioned, the minks (*n* = 5/group) were orally dosing or IP injected with T-2 toxin at 0, 0.05, and 0.25 mg/kg bw in 100 µL PBS. Following intramuscular administration, the animals were anesthetized with 10 mg/kg bw ketamine at 0, 60, 120, and 180 min intervals. Blood was collected via cardiac puncture using an EDTA vacuum and centrifuged for 10 min (1000× *g*, 4 °C) to plasma for GLP-1 and GIP (Phoenix Pharmaceuticals, Mannheim, Germany) ELISA examination.

Study 3: Effects of brain–gut peptide receptor inhibitor on T-2 toxin-induced emesis

To determine whether GLP-1R inhibitor Exendin9–39 could abate T-2 toxin-evoked emetic episodes, the minks were first given 1 mg/kg bw Exendin9–39 upon oral exposure. To estimate whether GIPR inhibitor Pro3GIP could attenuate T-2 toxin-induced vomiting, the minks were first gavaged with 0.5 mg/kg bw Pro3GIP. After that, the minks were fed for 30 min and provided with 0.25 mg/kg bw T-2 toxin upon oral or IP dosing. GLP-1 and GIP were also IP injected into the minks at 0.025 and 0.1 mg/kg bw as positive control. Emetic episodes including retch and vomit were recorded over the subsequent 6 h.

Study 4: Roles of CaSR and TRP channel on T-2 toxin-induced emesis and brain–gut peptides

To assess the effect of NPS or RR on T-2 toxin-induced emetic response, fasted minks (*n* = 5) were first orally gavaged with NPS (1, 2.5, and 5 mg/kg bw) or RR (0.5, 1, and 2.5 mg/kg bw) in 1 mL vehicle and provided 50 g feed immediately. To evaluate the combined effects of NPS and RR on T-2 toxin-induced emetic responses, fasted minks (*n* = 5) were first gavaged with 2.5 mg/kg bw NPS, 1 mg/kg bw RR, or both in 1 mL vehicle, and then provided 50 g of food. After 30 min, the minks were gavaged with 0.25 mg/kg bw T-2 toxin and monitored for emesis over the next 6 h.

To learn the dose–response impact of NPS and RR on T-2 toxin-induced brain–gut peptide release, fasted minks (*n* = 5) were orally gavaged with NPS (1, 2.5, and 5 mg/kg bw) or RR (0.5, 1, and 2.5 mg/kg bw) in 1 mL vehicle. To evaluate the combined effects of NPS and RR on T-2 toxin-induced brain–gut peptide release, fasted minks (*n* = 5) were first orally gavaged with 2.5 mg/kg bw NPS and/or 1 mg/kg bw RR in 1 mL vehicle or vehicle alone, respectively. After 30 min, the minks were gavaged with 0.25 mg/kg bw T-2 toxin. Upon experiment termination 2 h later, the minks were anesthetized with 10 mg/kg bw ketamine. Blood was collected via cardiac puncture using EDTA vacuum and centrifuged 10 min (1000× *g*, 4 °C) to plasma for GLP-1 and GIP (Phoenix Pharmaceuticals, Mannheim, Germany) ELISA examination.

### 5.3. Statistics

Data were calculated by SigmaPlot (Jandel Scientific; San Rafael, CA, USA) except for the emetic dose (ED), which was calculated through SAS using the Proc Probit method. Specific statistical methods were carried out as described in the figure legends, and differences were considered statistically significantly at *p* < 0.05.

## Figures and Tables

**Table 1:** Comparison of emetogenic potentials upon oral and IP exposure to T-2 toxin.

Exposure Route	Dose (mg/kg bw)	Incidence (Responding/Tested)	Latency (min) ^A,B^	Duration (min) ^A,B^	Emetic Episodes ^C^		
					Retch	Vomit	Total
Oral	0	0/5	-	-	0 ± 0	0 ± 0	0 ± 0
	0.002	0/5	-	-	0 ± 0	0 ± 0	0 ± 0
	0.01	0/5	-	-	0 ± 0	0 ± 0	0 ± 0
	0.05 *	4/5	28 ± 1 ^a^	63 ± 8 ^a^	72 ± 8	12 ± 3	84 ± 11
	0.25 *	5/5	19 ± 5 ^a^	124 ± 13 ^b^	132 ± 21	30 ± 8	162 ± 29
IP	0	0/5	-	-	0 ± 0	0 ± 0	0 ± 0
	0.002	0/5	-	-	0 ± 0	0 ± 0	0 ± 0
	0.01	0/5	-	-	0 ± 0	0 ± 0	0 ± 0
	0.05	2/5	32 ± 4 ^a^	48 ± 7 ^a^	34 ± 6	7 ± 2	41 ± 8
	0.25 *	5/5	25 ± 6 ^a^	161 ± 11 ^b^	151 ± 20	43 ± 7	194 ± 27

^A^ Average of positive responders only. ^B^ If animals did not elicit emetic episodes, latency and duration are displayed as “-”. ^C^ Average of both non-responders and responders. Data are mean ± SEM. * indicates significant differences at *p* < 0.05 for incidence, retch, vomit, and total emetic episodes. Different letters within a column indicate significant differences at *p* < 0.05.

## Data Availability

Not applicable.
